# Supplementary material for: Structural basis of sequence-specific RNA recognition by the antiviral factor APOBEC3G
Source: Nat Commun. 2022 Dec 5;13:7498. doi: 10.1038/s41467-022-35201-9 (PMC9722718; doi:10.1038/s41467-022-35201-9)

Supplementary Information

Structural basis of sequence-specific RNA recognition by the antiviral factor APOBEC3G

Hanjing Yang[1], Kyumin Kim[1], Shuxing Li[1,4], Josue Pacheco[1], Xiaojiang S. Chen[1,2,3,4,*]

[1] Molecular and Computational Biology, Departments of Biological Sciences and Chemistry: [2] Center of Excellence in NanoBiophysics; University of Southern California, Los Angeles, CA 90089, USA. [3] Genetic, Molecular and Cellular Biology Program, Keck School of Medicine, [4] Norris Comprehensive Cancer Center, University of Southern California, Los Angeles, CA 90033, USA.

This PDF file contains:

- Supplementary Table 1
- Supplementary Fig. 1 - 10
- Uncropped gel images of Supplementary Fig. 1a, 1b, and 2b

Supplementary Table 1. RNA used in this study.

RNA (Length)	Fluorophore	Sequence[a]
RNA1-top-NN (15 nt)		UUNNCGCUGCGGUGG
RNA1-bottom (11 nt)		CCACCGCAGCG
5'FAM RNA1-bottom (11 nt)	5'-6-FAM	CCACCGCAGCG
RNA2-top (11 nt)		CGCUGCGGUGG
5'FAM RNA2-top (11 nt)	5'-6-FAM	CGCUGCGGUGG
RNA2-bottom-NN (17 nt)		CCACCGCAGCGNNUUUU
RNA3-NN (10 nt)		UUUUNNUUUU
5'FAM RNA3-NN (10 nt)	5'-6-FAM	UUUUNNUUUU
RNA1-AA_{xtal}-top (18 nt)		UUAACGCUGCGGCCUUUU
RNA1-AA_{xtal}-bottom (18 nt)		UUAACCGCAGCGCCUUUU
RNA2-AA_{xtal}-top (14 nt)		CCCGUGGGAAUUUU
RNA2-AA_{xtal}-bottom (14 nt)		CCCACGGGAAUUUU
RNA3-AA_{xtal} (=RNA3-AA, 10 nt)		UUUUAAUUUU
RNA3-GA_{xtal} (=RNA3-GA, 10 nt)		UUUUGAUUUU

[a]NN as specified in the text.

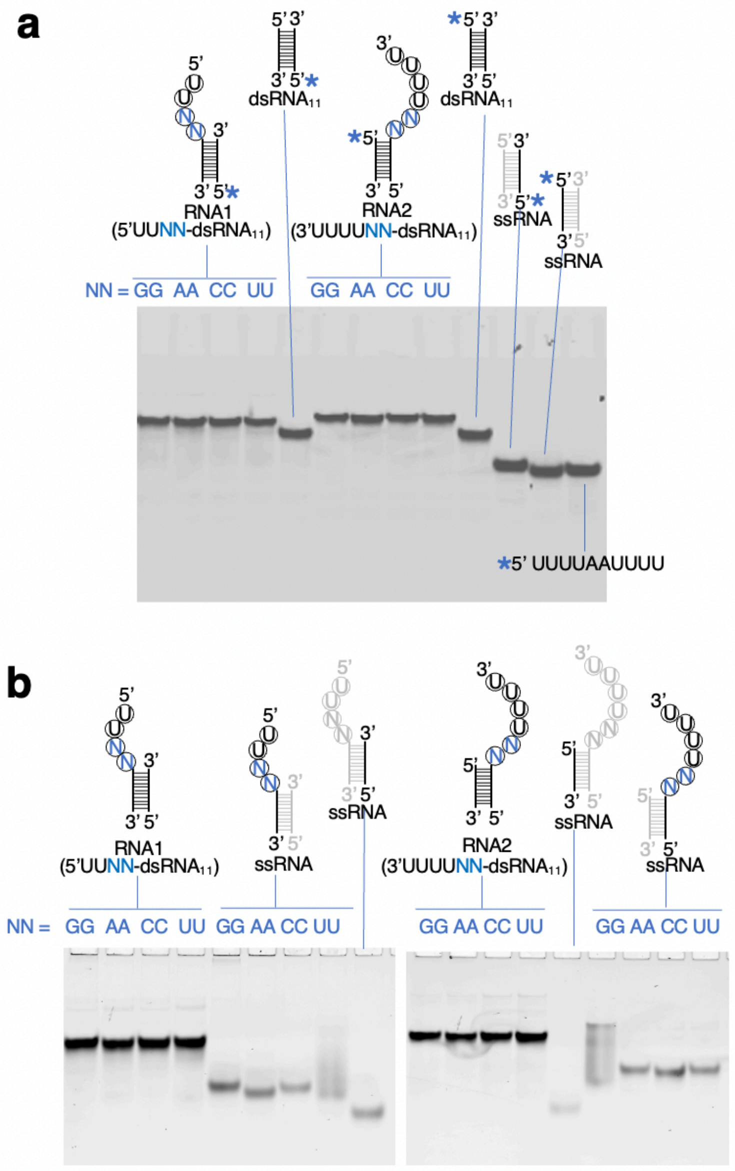

Supplementary Fig. 1. Formation of annealed dsRNA with overhangs verified by native gel electrophoresis. Dinucleotides NN = GG, AA, CC or UU. a Gel image of each synthesized 6-FAM labeled RNA strand annealed to its corresponding complementary RNA strand to form dsRNA with 5' or 3' overhang. Asterisk (*) marks the location of 6-FAM. $dsRNA_{11}$ represents an 11-bp double stranded RNA molecule (Supplementary Table 1). Two Annealed 6-FAM labeled $dsRNA_{11}$ molecules are used as size markers. b Gel images of each unlabeled RNA strand annealed to its corresponding complementary RNA strand to form dsRNA with 5' or 3' overhang. Gels were stained with SYBR™ Gold Nucleic Acid Gel Stain for detection of RNA. A dsRNA ladder (NEB) was used as size markers. These annealing experiments were done one time**.**

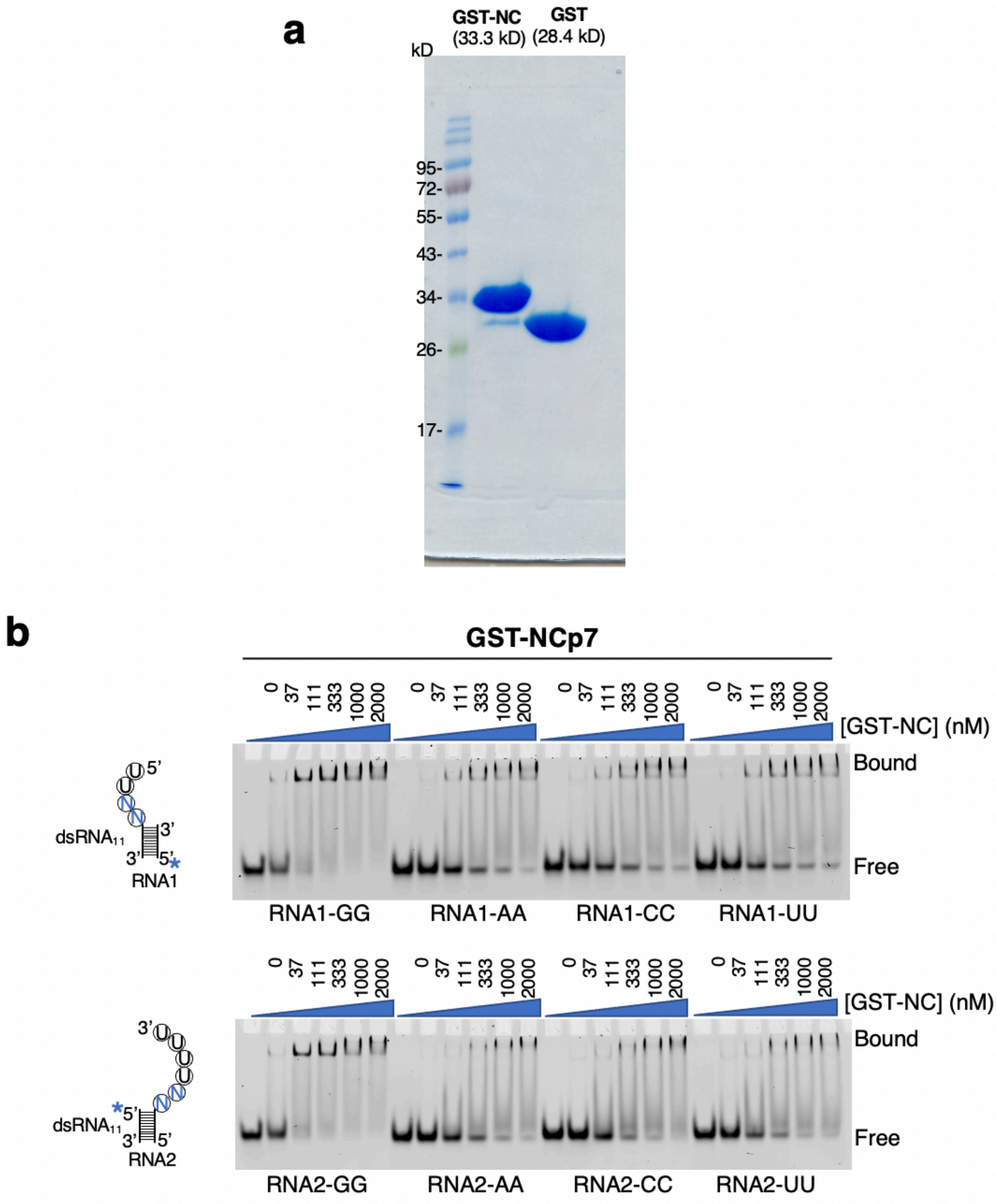

Supplementary Fig. 2 GST-NCp7 binding to various synthesized RNA molecules. **a** A gel image of purified GST-NCp7 and GST proteins. Each protein sample (~10 mg) was used with 12% SDS-PAGE. Purification of GST-NCp7 was done one time. **b** GST-NCp7 binding to RNA1-NN and RNA2-NN, where NN = GG, AA, CC, or UU. RNA at a fixed concentration of 10 nM was incubated with GST-NCp7 at various concentrations (0, 37, 111, 333, 1,000, and 2,000 nM). Representative EMSA gel images are shown. Asterisk marks the location of 6-FAM. $dsRNA_{11}$ represents an 11-bp double stranded RNA molecule (Supplementary Table 1). n = 3 independent experiments.

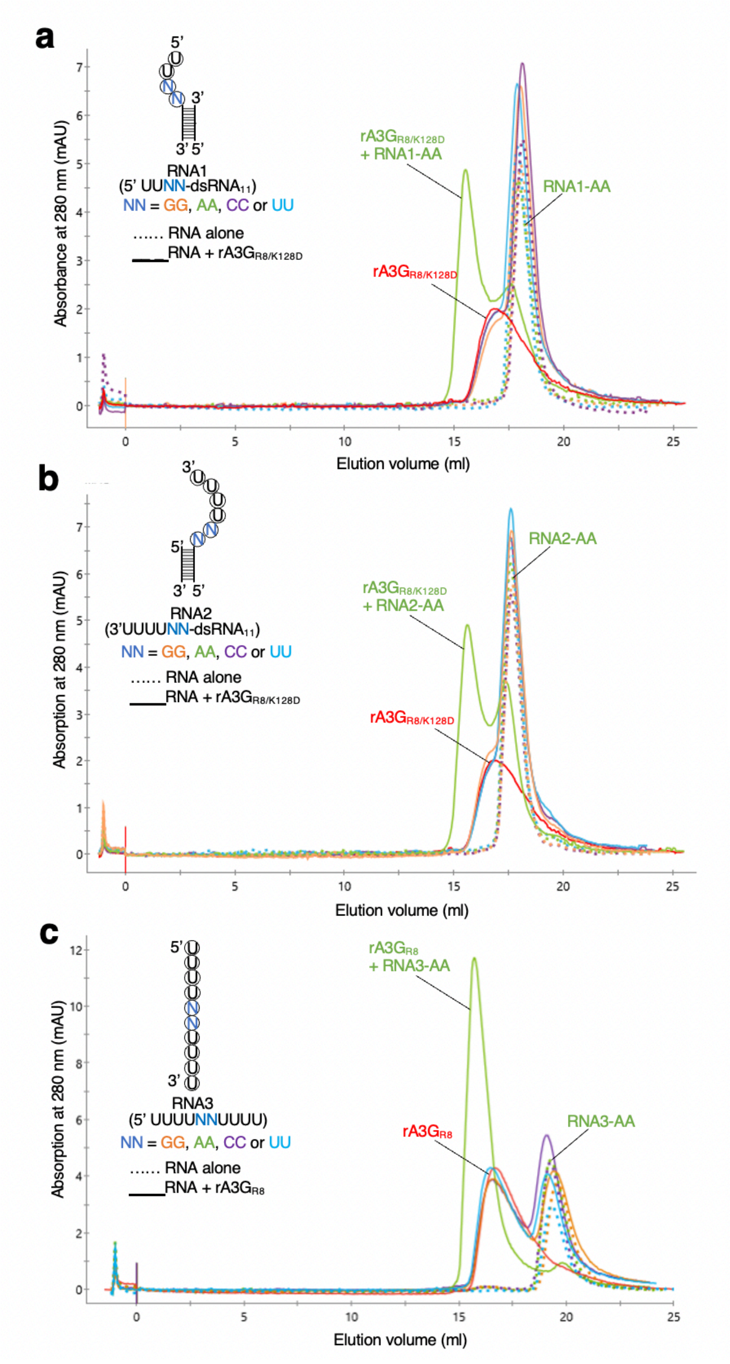

Supplementary Fig. 3 FPLC Superdex 200 Increase 10/300 GL chromatogram overlay of RNA alone, purified RNA-free rA3G alone, or their mixture, showing peak shift of the mixture with AA-containing RNA. (**a**) 5'-overhang dsRNA alone (NN = GG, AA, CC or UU, dotted lines), rA3G alone (solid red line), or their mixture (solid lines). (**b**) 3'-overhang dsRNA alone (NN = GG, AA, CC or UU, dotted lines), rA3G alone (solid red line), or their mixture (solid lines). (**c**) ssRNA alone (NN = GG, AA, CC or UU, dotted lines), rA3G alone (solid red line), or their mixture (solid lines). $dsRNA_{11}$ represents an 11-bp double stranded RNA molecule (Supplementary Table 1). Source data are provided as a Source Data file.

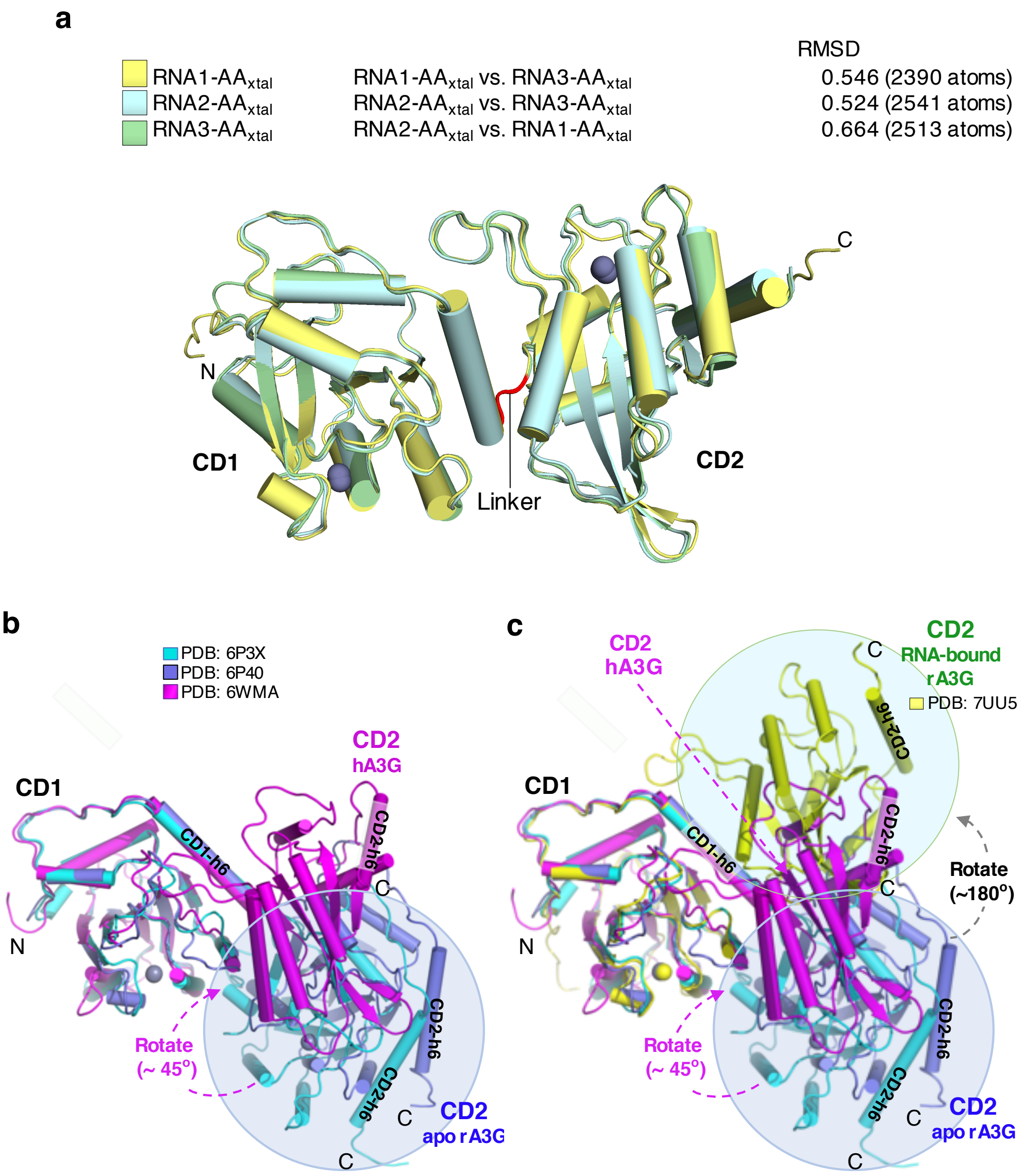

Supplementary Fig. 4 Comparison of the domain orientations of the previously reported structures of A3G (rA3G and hA3G) and the RNA-bound structures of rA3G presented here. (**a**) Alignment of all three different structures of rA3G-RNA complexes described in this report, showing essentially the same rA3G conformation with the same CD1-CD2 domain orientation. (**b**) The alignment of CD1 domains between all three available apo structures of rA3G and hA3G, with CD1-h6 as a marker of overlap. The positions of the CD2 of apo rA3G structures (in cyan: 6P3X and blue: 6P40) differ a few degrees from each other. The CD2 of hA3G structure (in magenta: 6WMA) turns about 90° and rotate about -45° from the CD2 of apo rA3G. (**c**) When the CD1 of the RNA-bound rA3G structure (in yellow, PDB ID: 7UU5) is superimposed with the CD1 domains of all the previously reported structures as shown in panel-a, the CD2 turns ~135° and rotates about 180° from the CD2 of apo rA3G. The N- and C- termini are labeled.

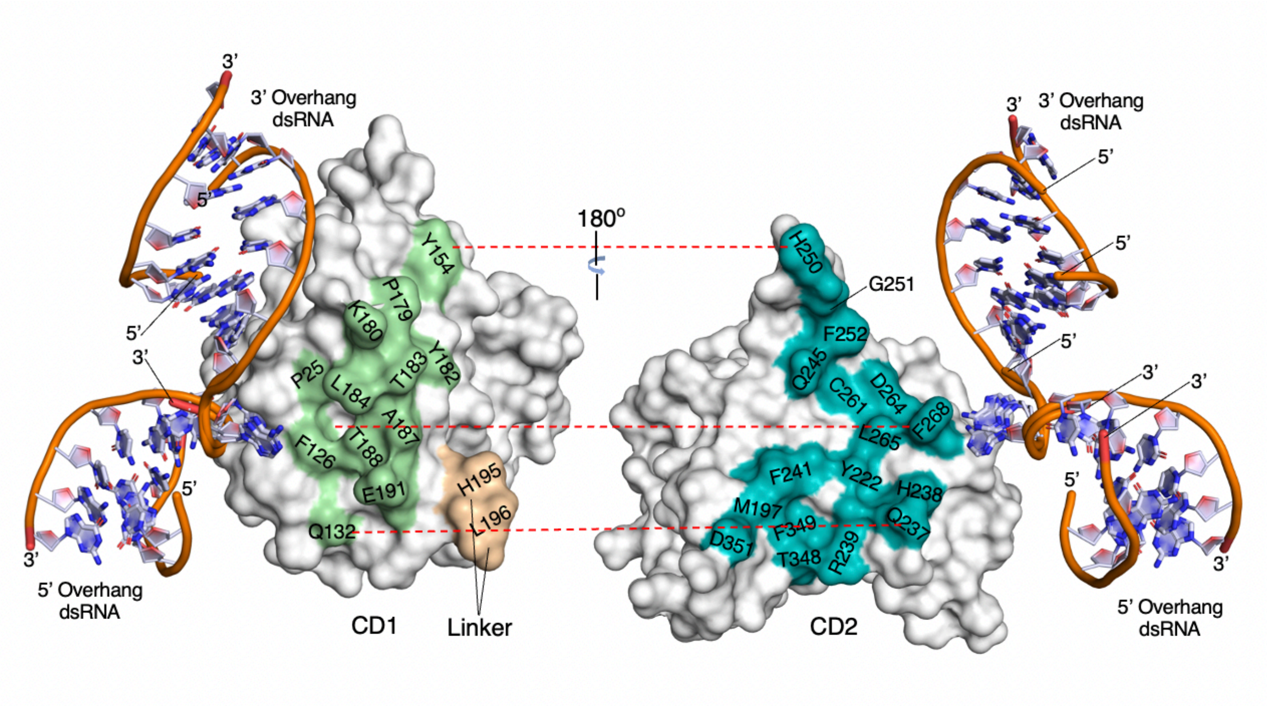

Supplementary Fig. 5 Surface representations of the interface residues with buried surface area larger than 10 Å^2. Three contacting points are selectively indicated by dash lines (in red). 5'- and 3'-overhang dsRNA bound on the surface of rA3G are shown as the orientation markers.

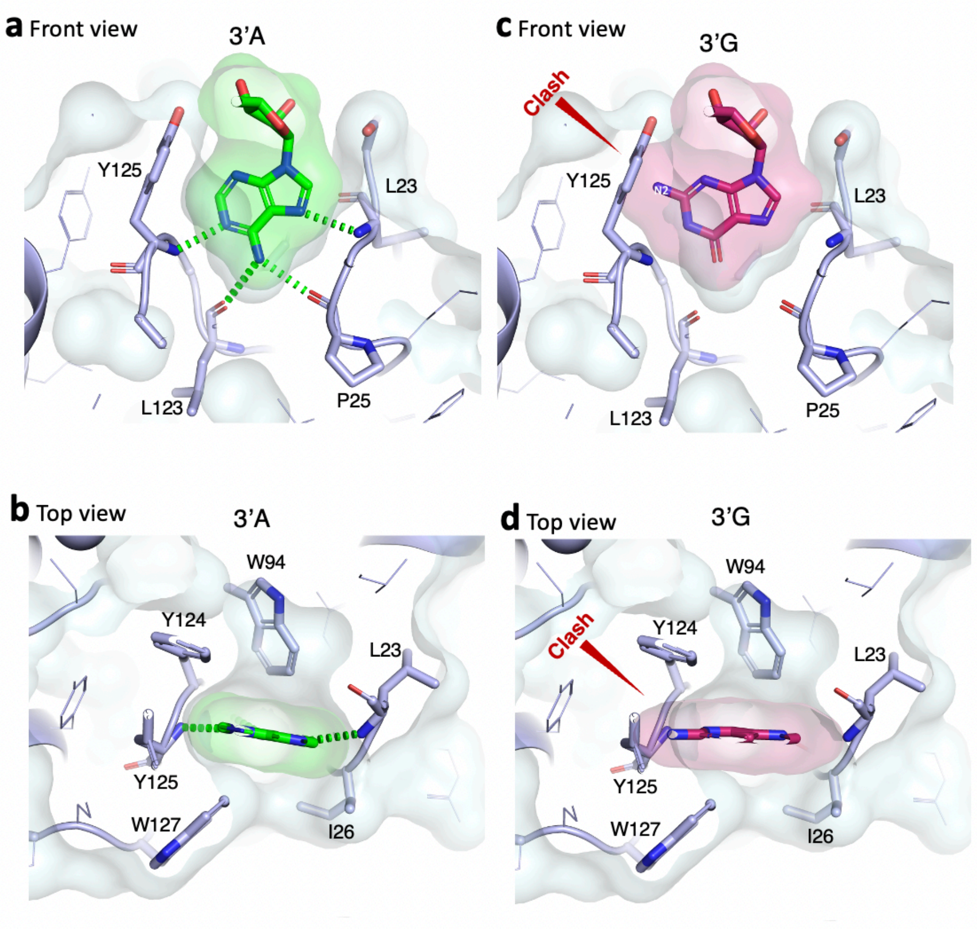

Supplementary Fig. 6 *In silico* analysis of substitution of 3’A with 3’G. (**a-b**) A 3’A fits in the pocket (PDB ID: 7UU4). Local surface electrostatic potential around N6 of 3’A is negative and N6 forms two H-bonds with two main-chain carbonyl groups of P25/L123. (**c-d**) A 3’G doesn’t fit in the pocket. Local surface electrostatic potential around O6 of 3’G is negative, rendering it unfavorable for 3’G binding. There is also a steric clash between N2 of 3’G and the aromatic ring of Y125.

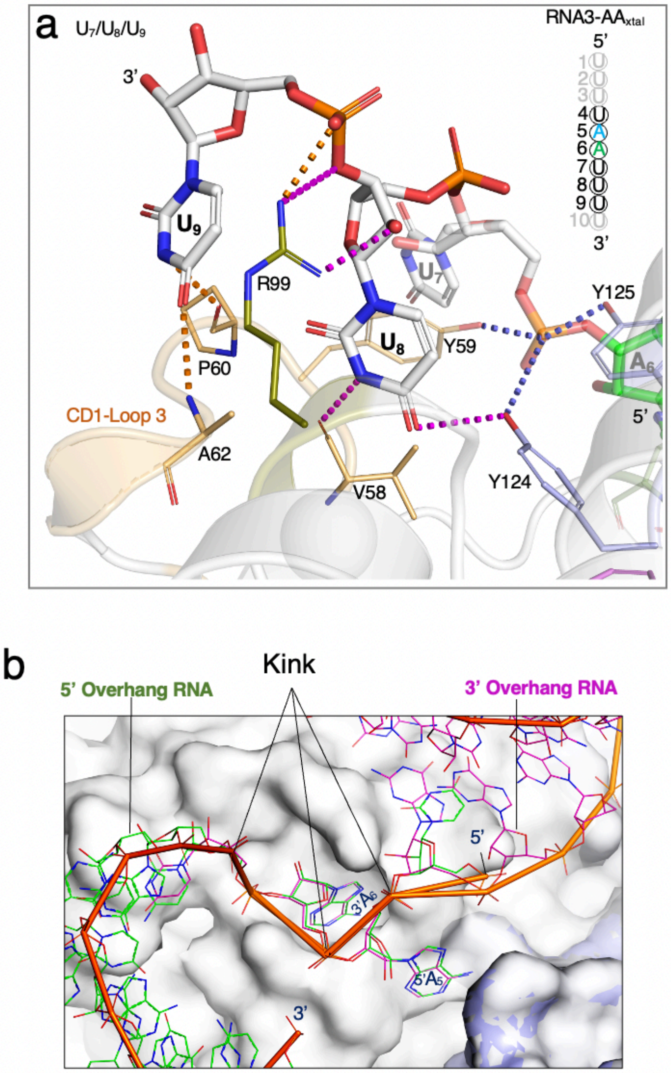

Supplementary Fig. 7 (**a**) Contacts between rA3G$_{R8/E259A}$ and U_7/U_8/U_9 in the co-crystal structure with ssRNA (PDB ID: 7UU4). Contacts between U_7 and rA3G: three H-bonds (colored in slate) are formed between its 5' phosphate oxygen OP1 (or 3' phosphate oxygen of A_6) and three tyrosine residues Y59 (on loop 3, 2.5 Å), Y124 and Y125 (both on loop 7, 3.4 Å and 2.6 Å). U_8 is located close to the zinc center of CD1. Four H-bonds are formed (colored in magenta): V58 backbone to the base (2.9 Å), Y124 side chain to the base (3.6 Å), R99 side chain to the RNA specific O2' (3.0 Å) and sugar (O3', 3.8 Å). U_9 sits in between CD1-loop 3 and the residue R99. Three H-bonds are formed (colored in orange): P60 backbone to the base (3.1 Å), A62 backbone to the base (3.1 Å), and R99 side chain to the phosphate backbone (2.9 Å).
(**b**) The sharp RNA kinks on the side of each adenosine of the AA dinucleotide (or 5'A_5, 3'A_6 as numbered in the ssRNA bound structure) bound by the pocket and the interface groove on rA3G**.**

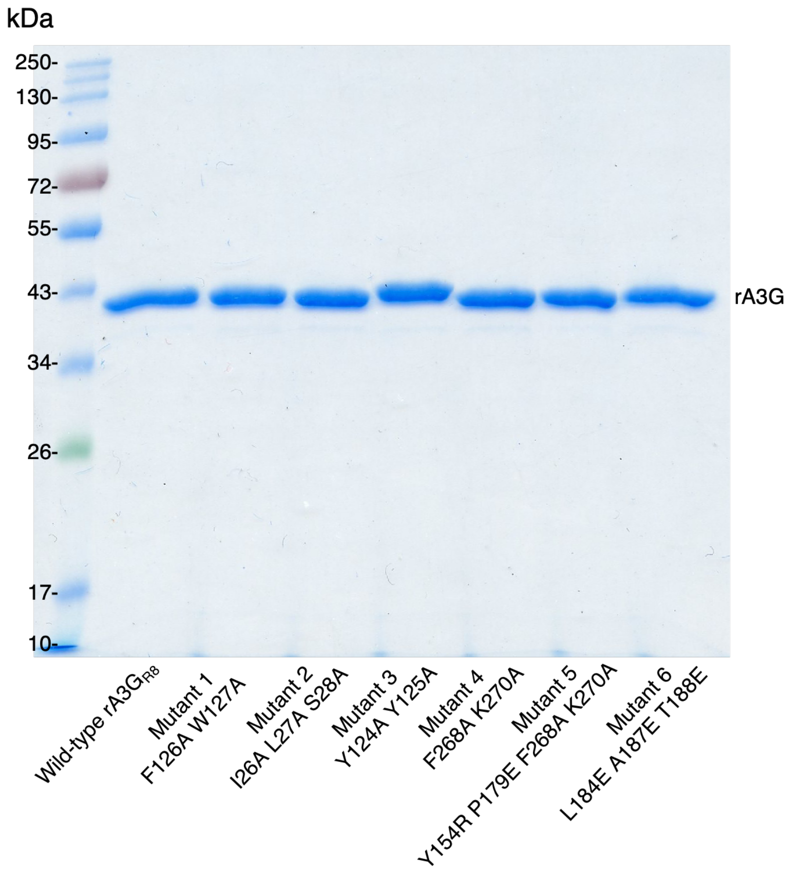

Supplementary Fig. 8 SDS-PAGE of purified individual rA3G mutant 1-6 protein samples used for RNA binding assays as shown in Fig. 7j. Each sample was prepared with 2 µl at 10 µM concentration for 12% SDS-PAGE. A gel image is shown after Coomassie blue staining.

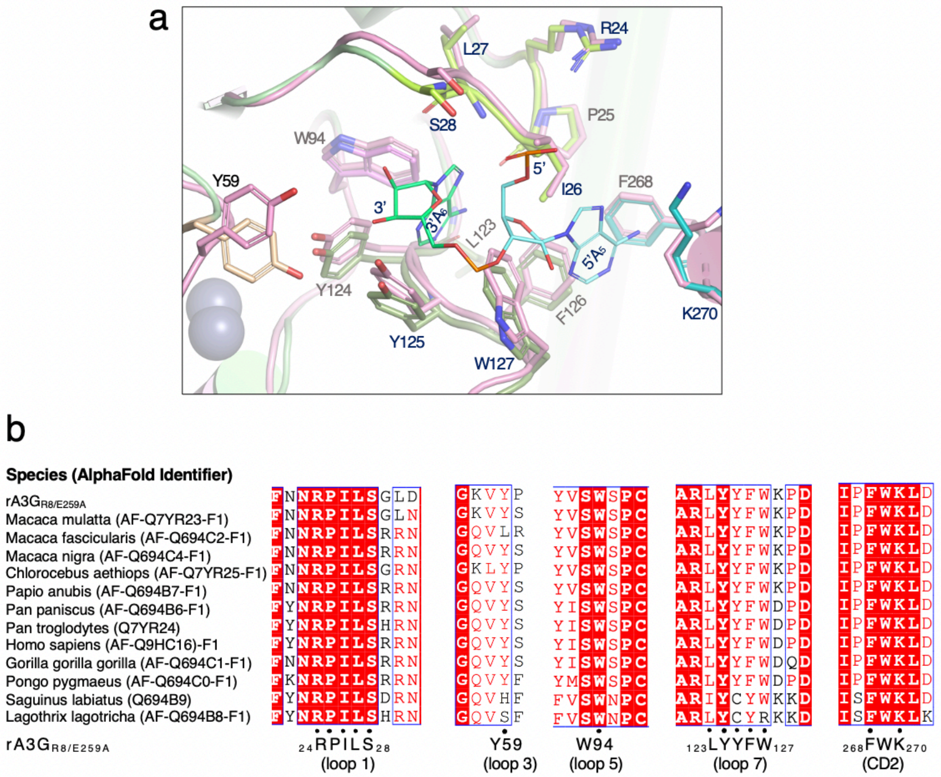

Supplementary Fig. 9 A3G homolog alignment. (**a**) Structure alignment of rA3G (in green) binding pockets for the ssRNA -AA- dinucleotide with that of modeled hA3G structure (in pink) reveals identical residues on CD1 and CD2 to interact with the AA dinucleotide 5’A_5 and 3’A_6 of the bound RNA (cyan). (**b**) Primary protein sequence alignment of twelve primate A3G homologs, showing the conserved residues forming the “pocket” and the “groove” for binding the AA dinucleotide on both CD1 and CD2.

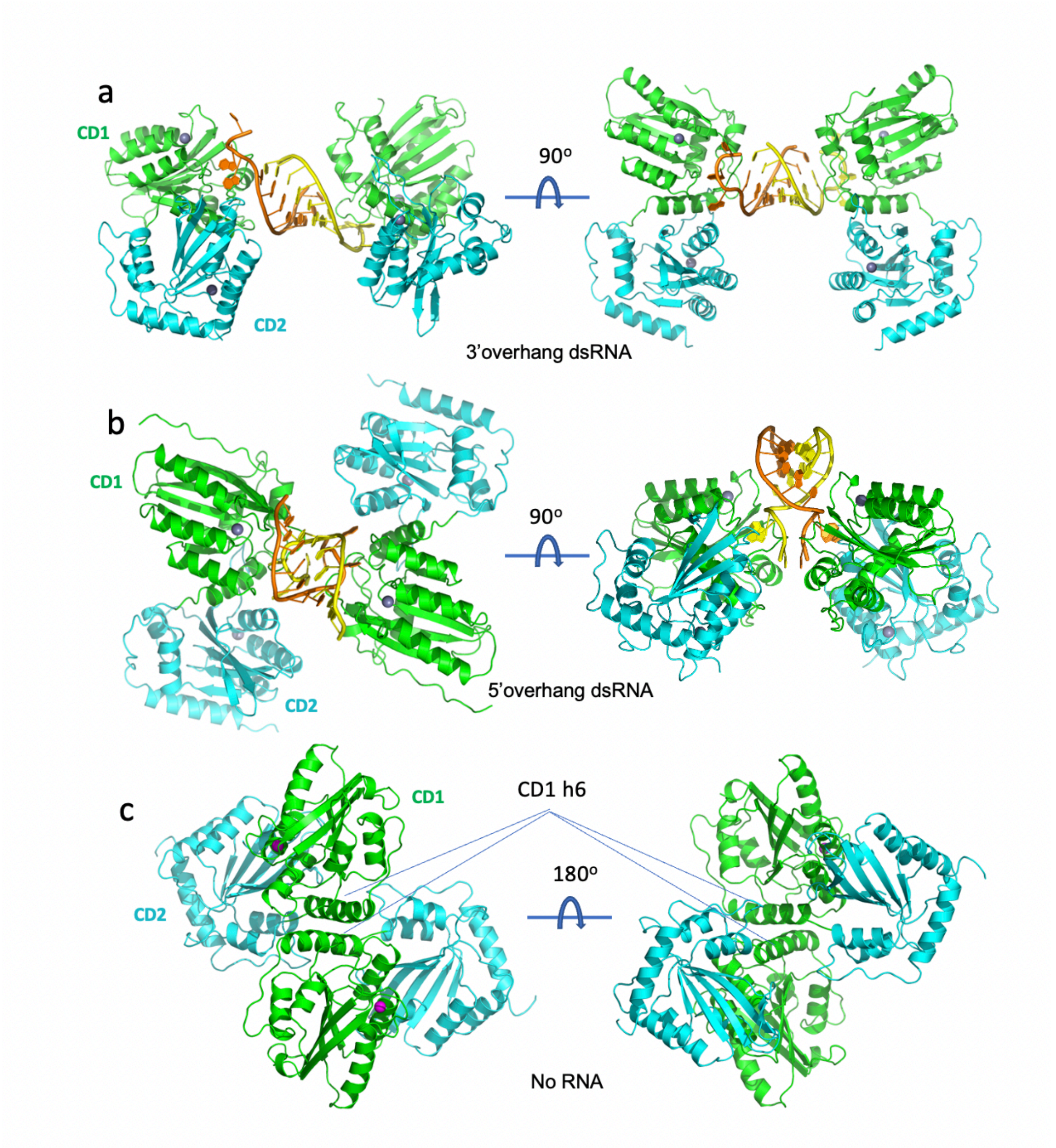

Supplementary Fig. 10 Different types of rA3G dimers. The CD1 (green) and CD2 (cyan) are labeled for one rA3G in each dimer form. **(a, b)** two different RNA-mediated dimers of rA3G when binding to the 3'overhang dsRNA (a) or 5'overhang dsRNA (b) containing AA dinucleotide. In 3'-overhang dimer in panel-**a**, the distance between the two rA3G in a dimer will depend on the length of the dsRNA region. In the 5'-overhang dimer in panel-**b**, the distance between the two rA3G in a dimer is not dependent on the dsRNA length. **(c)** The apo rA3G dimer is formed via CD1 h6-h6 interactions (PDB: 6P3X). In the RNA-bound forms, the CD1 h6 of rA3G is completely buried within the interface with CD2, thus excluding the CD1 h6-h6 dimerization observed for apo-rA3G.

Uncropped gel image of Supplementary Fig. 1a

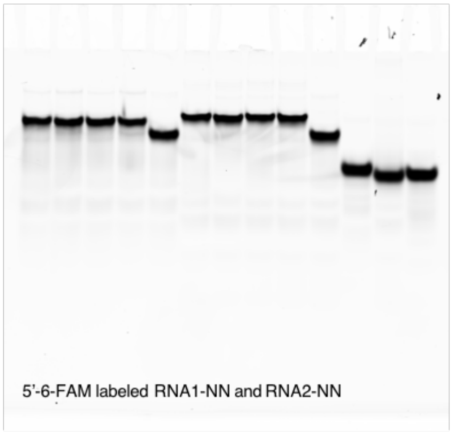

Uncropped gel images of Supplementary Fig. 1b

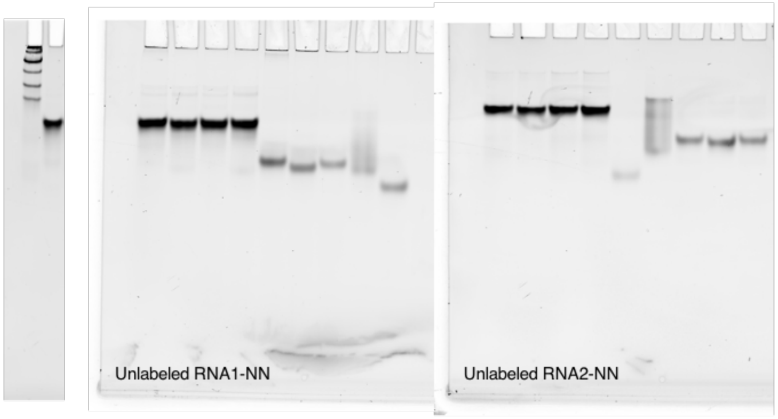

Uncropped gel images of Supplementary Fig. 2b

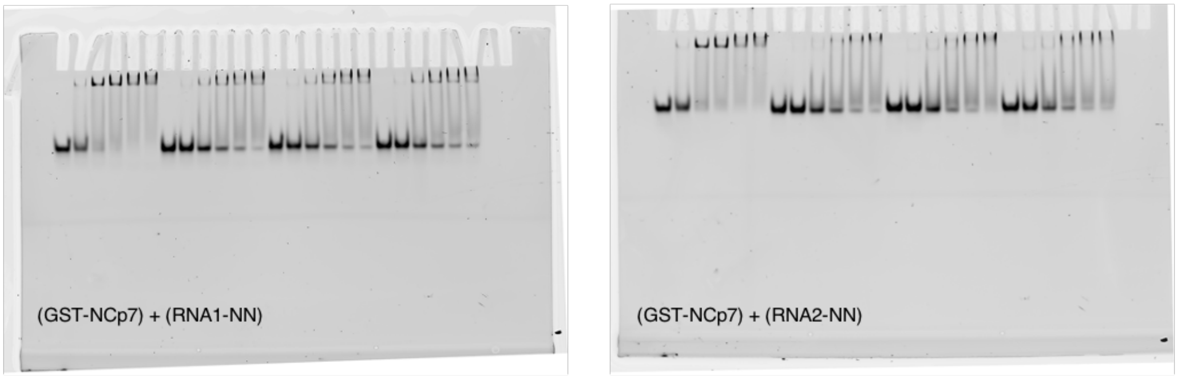

Supplement: Supplementary file 1 — Supplementary Information [file 41467_2022_35201_MOESM1_ESM.pdf]
